# Prospective study of exposure to smoking imagery in films and changes in susceptibility to smoking in a cohort of school students in Southern India

**DOI:** 10.18332/tid/178442

**Published:** 2024-05-28

**Authors:** Veena G. Kamath, Muralidhar M. Kulkarni, Asha Kamath, Sarah Lewis, Ilze Bogdanovica, Manpreet Bains, Jo Cranwell, Andrew Fogarty, Monika Arora, Deepika Bahl, Gaurang P. Nazar, Ashwath K. Naik, Kirtinath Ballal, Rohith Bhagawath, John Britton

**Affiliations:** 1 Department of Community Medicine, Kasturba Medical College, Manipal Academy of Higher Education, Manipal, India; 2 Department of Data Science, Prasanna School of Public Health, Manipal Academy of Higher Education, Manipal, India; 3 UK Centre for Tobacco and Alcohol Studies, University of Nottingham, Nottingham, United Kingdom; 4 Department for Health, University of Bath, Bath, United Kingdom; 5 HRIDAY – Promoting Sustainable Health, New Delhi, India; 6 Health Promotion Division, Public Health Foundation of India, Gurugram, India

**Keywords:** smoking susceptibility, smoking imagery, tobacco-free film rules, cohort study

## Abstract

**INTRODUCTION:**

India has unique tobacco-free film and TV rules designed to prevent tobacco uptake. In this study, we examined the potential influence of exposure to smoking imagery in regionally famous films, on susceptibility to smoke in teenagers enrolled in schools in a district in Southern India.

**METHODS:**

A longitudinal survey of students, in grades 6 to 8 at baseline in 2017 and grades 7 to 9 one year later in 2018, ascertained prospective incident susceptibility to smoking during the study period in relation to baseline exposure to 27 locally popular films with actual or implied smoking imagery.

**RESULTS:**

We analyzed linked data from 33676 participants, and 3973 (11.8%) of the adolescents reported incident susceptibility. There was a significant increase in susceptibility to smoking with increasing exposure at baseline to smoking imagery in films on univariable analysis, highest tertile of exposure relative to no exposure (OR=1.4; 95% CI: 1.0 –2.1, p_trend_<0.001), and this trend remained significant (p=0.022) after mutual adjustment for recognized confounders, highest vs no exposure (AOR=1.3; 95% CI: 0.9–1.8). We found no statistically significant association between exposure to tobacco-free film rules and change in susceptibility.

**CONCLUSIONS:**

Prospectively, watching films featuring smoking imagery increases adolescents' vulnerability to smoking. Further research revealed no difference in susceptibility change between youth who saw partially compliant films and those who watched non-compliant films. Our findings, thus, underscore the need to incorporate comprehensive approaches to prevent the inclusion of smoking imagery in films.

## INTRODUCTION

Smoking is the largest preventable cause of disease and death across the world, with around 80% of smoking-attributable deaths occurring in Low- and Middle-Income Countries^1^. Adolescence and young adulthood are the times when most smokers initiate tobacco use^2^, and in 2019 there were 155 million young people (aged 15–24 years) who were tobacco smokers, globally^3^. According to the Global Adult Tobacco Survey (GATS) II, the prevalence of current smoking among adults in India in 2017 was 10.7%, down from 14.0% in 2009^4^. However, among adolescents (aged 13–15 years), the prevalence of smoking fell less than one percent, from 2009 to 2019^5^. This more modest decrease among adolescents suggests that the extensive package of tobacco control measures, introduced over this period in India^6-8^, needs to prioritize the adolescent population.

Smoking initiation has a complex etiology that includes individual, interpersonal, and environmental risk factors^9^. Exposure to smoking imagery in films is proven, but many countries neglected avoidable exposure-related risk factors for smoking uptake among adolescents^10,11^. To address this exposure, the Government of India enacted in 2012 tobacco-free film and TV rules that mandate all films to have audio-visual disclaimers and 30-second long health spots in the beginning and middle of the film, and to show on-screen static warning messages whenever smoking imagery is depicted^12^.

We have previously reported evidence that the tobacco-free film and TV rules in India probably attenuate the impact of smoking imagery on smoking in young people^13,14^, but to our knowledge, no studies have explored the effect of this legislation on measures of susceptibility to future smoking among adolescents. We report a prospective analysis of change in susceptibility to smoking in relation to exposure to smoking imagery in films in the cohort of adolescents aged 11–15 years studied in our earlier studies^13,14^.

## METHODS

Data for the study were taken from a previously reported one-year prospective cohort study of the relation between exposure to tobacco imagery in film and incident smoking in 39282 students in grades 6, 7, and 8 (ages 10–15 years) in government (n=713), government-aided (n=265), and privately-funded schools (n=232) in Udupi district of Karnataka State in India. Participants were surveyed at baseline in 2017 and again in 2018^13^. Questionnaire data from the two surveys were linked with the help of the unique enrolment number given to every child in formal education in Karnataka state. Considering high rates for school attendance, we included only those children who were present on the arranged study day; if, for any reason (for example, heavy monsoon rains), fewer than 80% of students were in attendance, the survey was rescheduled. Ethics approvals were granted by the Manipal Academy of Higher Education [MAHE EC/012/2017], Nottingham University [OVS200317] Ethics committees, Centre for Chronic Disease Control [CCDC_IEC_11_2018], and the Indian Health Ministry’s Screening Committee [HMSC 2017-0460].

### Questionnaire design and study variables

As described in our earlier publication^13^, the questionnaire elicited information on current and past use of cigarettes, beedis, and a range of other smoked tobacco including cigars, cheroots, chillum, chutta, and rolled cigarettes^15^, with a frequency of use (never, ever but not now, less than once a week, once a week, daily) using questions adapted from the Global Youth Tobacco Survey^16^, the UK Smoking, Drinking and Drug Use (SDD) survey^17^, and HRIDAY’s Mobilizing Youth for Tobacco-Related Initiatives in India (MYTRI) project^18^. The questionnaire included questions on age, gender, religion, academic grades in the past year, expectation of academic achievement, parental education and occupation, family and peer smoking, self-esteem, and rebelliousness^19,20^. We measured socioeconomic status by asking a question on ownership of household goods, grouping participants into quintiles of family wealth^21^. Smoking susceptibility among never smokers (n=3973) was elicited using four previously validated questions: ‘Do you think you will try a cigarette soon?’ (yes/no); ‘If one of your best friends were to offer you a cigarette, would you smoke it’ (definitely yes, probably yes, probably not, definitely not); ‘Do you think you will smoke a cigarette at any time during next year?’ (definitely yes, probably yes, probably not, definitely not); and ‘Do you think you will smoke any time once you go to college?’ (definitely yes, probably yes, probably not, definitely not)^22,23^. Questions on the smoking policies adopted by the respondents’ schools and in the family home were also included.

Questions on exposure to tobacco imagery in films asked students in 2017 whether they had seen any of 27 of the most popular films in Karnataka in 2015 and 2016 previously demonstrated to include smoking imagery^24^. The identification and content coding of these films have been reported in detail in our previous publication^24^, but in brief, we used national and local box office and distributor takings to identify 47 of the most popular films in Karnataka in the given years and used 5-minute interval coding to provide semi-quantitative estimates of tobacco content in them. We then selected the 27 films found to contain smoking imagery for inclusion in the questionnaires and asked all participants to report whether they had seen each of these films. Total exposure to tobacco imagery was estimated by summing the number of 5-minute coded intervals containing tobacco imagery contained in each of the films and assuming one complete viewing per film reported as seen^24^. The questionnaire was refined after pilot testing in a school in the neighboring district before the survey.

### Data analysis

Data were extracted from questionnaires into Microsoft Excel using Optical Mark Reader scanning and transferred into STATA 9.2 software for analysis. Questionnaires from the two surveys were linked using each student’s unique enrolment number and by using other questionnaire responses (for example, age, school, and grade) to confirm matches in the event of minor coding discrepancies. Standardized definitions were used to define the never smokers, ever smokers, current smokers, and incident smokers^13,14^. Never smokers were defined in both surveys as those who reported that they had never smoked, not even a puff or two, currently or anytime in the past. Ever smoking was defined as any reported smoking of any tobacco product, currently or in the past, and incident smoking was the reporting of ever smoking in 2018 (Year 2) among participants who reported that they had never smoked in 2017 (Year 1). Non-susceptibility to smoking was defined as answering ‘no’ to the relevant four questions described above, while any other combination was defined to be susceptible. We defined incident susceptibility as the occurrence of susceptibility to smoking in non-smoking Year 2 students who were non-susceptible never smokers in Year 1.

Associations between change in susceptibility status (from non-susceptible to susceptible) and categorical variables, were investigated using logistic regression to estimate the effects of potential explanatory variables measured in Year 1 on the likelihood of change in susceptibility status. We first report unadjusted models among those who were non-susceptible never smokers at baseline and then adjust this model for known and potential confounding variables that were found to be significant in the univariable analysis. We then tested the effect of exposure to films containing smoking imagery before and after correcting for these independently significant variables, first treating smoking imagery exposure as a binary (yes or no) exposure and then, given that almost all children were exposed to some degree, as a graded exposure with four categories (none, and ordered tertiles) of exposure to smoking intervals in films.

In a post hoc analysis, we then explored the effects of exposure to film smoking intervals in relation to the degree of film compliance with tobacco-free film and TV rules, adjusting for the same potential confounders. Films were categorized as compliant, partially compliant, or non-compliant in relation to audio-visual (AV) disclaimers and health spots at the start and middle of the films. On-screen health warnings were analyzed in two binary categorizations: health warnings present but not necessarily fully compliant with COTPA rules on color and legibility or not, and health warnings present and fully compliant with COTPA rules or not. We then ran a single model multinomial logistic regression to explore the impact of watching films with varying degrees of compliance to tobacco-free film and TV rules on change in the smoking susceptibility status from susceptible to non-susceptible, from non-susceptible to susceptible, and sustained non-susceptible. Each result in multinomial regression was modeled in relation to the baseline outcome group, which in this study was defined as being non-susceptible. The odds ratios (OR) and their respective 95% confidence intervals are presented in the tables. A chi-squared test for trends was used to assess the linear trend in the OR. All tests were two-tailed, and the level of significance was 5%.

## RESULTS

**Figure 1:**
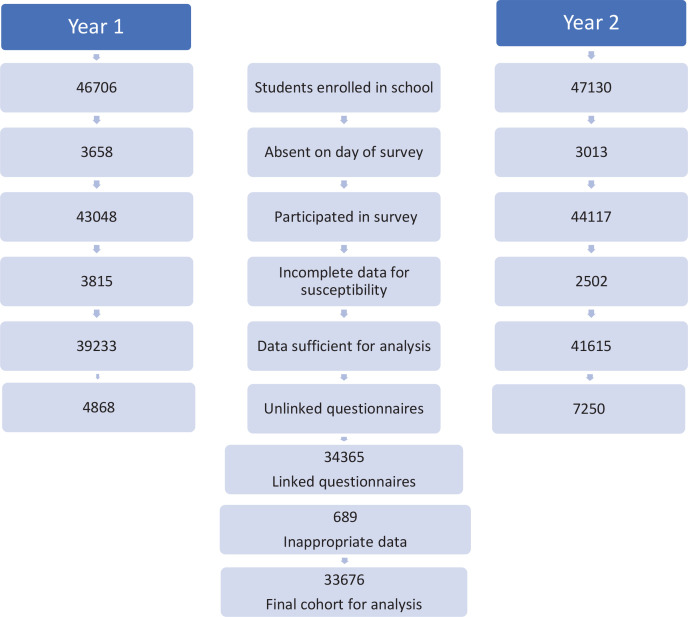
Flow chart of students aged 10–15 years, who participated in the two surveys, 2017–2018 (N=33676)

In Year 1, as previously reported, 39282 of 46706 (84%) students enrolled in grades 6 to 8 in 914 schools provided questionnaire responses sufficiently complete for analysis^13^, while in Year 2, a total of 47130 students were enrolled in grades 7 to 9 in 904 schools (14 schools had closed, and four new schools opened between studies), of whom 41615 (88%) provided responses sufficiently complete for analysis^14^. We were able to link Year 2 data with Year 1 responses for 34365 students, and after excluding responses from 689 individuals with incompatible ever smoking responses, n=567 (positive in Year 1 and negative in Year 2) and excluding n=122 ever smokers in Year 1, we analyzed responses from 33676 participants who had provided valid questionnaire responses in both surveys and a single missing observation on the outcome variable was excluded from the analysis. A more complete breakdown of these figures is presented in Figure 1.

**Figure 2:**
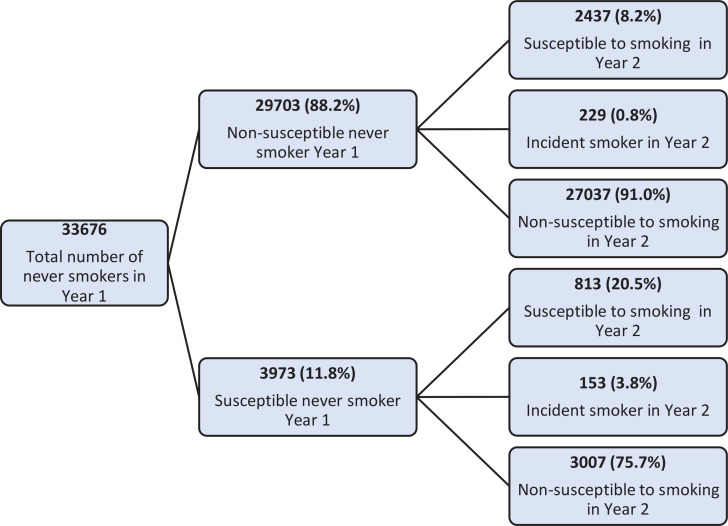
Flow chart depicting the change in susceptibility status from Year 1 to Year 2 of students aged 10–15 years, 2017–2018 (N=33676)

In Year 1, a total of 29703 (88.2%) never smoking students were categorized as non-susceptible and 3973 (11.8%) as susceptible to smoking in the future. Of those who were non-susceptible at baseline, 2437 (8.2%) became susceptible, 229 (0.8%) became smokers, and 27037 (91%) did not change their status in Year 2. In Year 1, among susceptible non-smokers, 3007 (75.7%) became non-susceptible, 813 (20.5%) remained susceptible, and 153 (3.8%) initiated smoking (Figure 2). Almost all participants (n=29240; 98.4%) reported having seen at least one of the 27 films containing smoking imagery listed in the baseline questionnaire.

**Figure 3:**
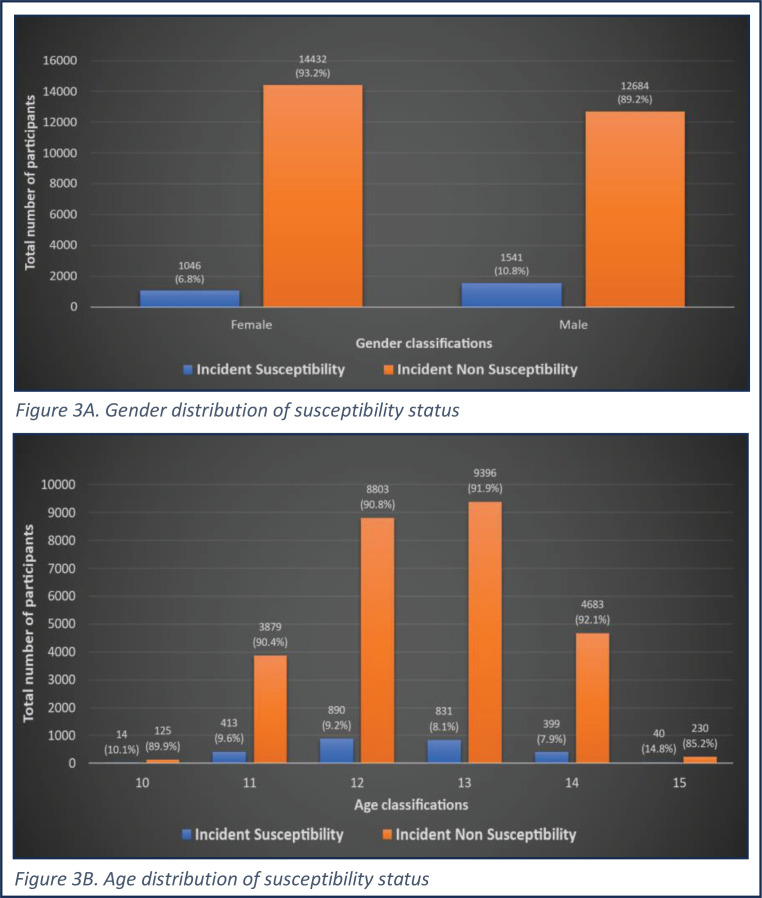
Smoking susceptibility status by age and gender among study participants aged 10–15 years, 2017–2018 (N=33676)

In univariable analysis, the onset of susceptibility during the study period (2017–2018) was significantly associated with increasing age and was more likely among male participants (Figure 3), those attending government-funded or part-funded schools, those who were non-Hindu, among those who had a father, siblings or friends who smoked, lived in a home where smoking is allowed, went to a school where they had observed smoking, whose parents were less educated, who were rebellious, reported low self-esteem, and had poor performance at school (Table 1). In a mutually adjusted model retaining independently significant associations, the incidence of smoking susceptibility was related to age, being male, non-Hindu, attending public or part-funded schools, living in a home where smoking is allowed, having father or friends who smoke, being rebellious and having poor performance at school (Table 1). In a model mutually adjusted for these effects, there was no statistically significant association between exposure to smoking in films, expressed as a binary variable, and the onset of susceptibility (OR=1.3; 95% CI: 0.9–1.8, p=0.220). However, when exposure to tobacco imagery in films was included as a graded variable with exposure categorized into tertiles of the number of intervals containing smoking imagery in the films, relative to no exposure, there was a significant trend of increasing susceptibility with increasing tertile of exposure, with an odds ratio for the highest tertile relative to no exposure of 1.4 (95% CI: 1.0–2.1, p_trend_<0.001). This trend remained significant, though it was slightly weaker, after adjustment for confounders (OR for highest vs no exposure=1.3; 95% CI: 0.9–1.8, p_trend_=0.022). Additionally, there was no statistically significant association between watching movies that are not compliant with tobacco-free film and TV rules and the onset of susceptibility (OR=0.6; 95% CI: 0.3–1.3, p=0.183)

**Table 1:** Demographic and environmental associations with smoking susceptibility in the study participants aged 10–15 years, with univariable and mutually adjusted odds ratios, 2017–2018 (N=33676)

*Characteristics*	*Number (N=29703)*	*Incident susceptibility (N=2587) n (%)*	*Univariable OR (95% CI)*	*p*	AOR* (95% CI)	*p*
**Age** (years)				<0.001		<0.001
10 ®	139	14 (10.1)	1		1	
11	4292	413 (9.6)	1.0 (0.5–1.7)		1.0 (0.6–1.7)	
12	9693	890 (9.2)	0.9 (0.5–1.6)		0.9 (0.5–1.6)	
13	10227	831 (8.1)	0.8 (0.5–1.4)		0.8 (0.5–1.4)	
14	5082	399 (7.9)	0.8 (0.4–1.3)		0.8 (0.4–1.4)	
15	270	40 (14.8)	1.6 (0.8–3.0)		1.4 (0.7–2.7)	
**Gender**				<0.001		<0.001
Female ®	15478	1046 (6.8)	1		1	
Male	14225	1541 (10.8)	1.7 (1.5–1.8)		1.6 (1.5–1.8)	
**School locality**				0.346		
Rural ®	23918	2065 (8.6)	1			
Urban	5785	552 (9.0)	1.1 (1.0–1.2)			
**School type**				0.040		0.055
Private (public) ®	11388	973 (8.5)	1		1	
Government	12714	1078 (8.5)	1.0 (0.9–1.1)		1.0 (0.9–1.1)	
Aided (part funded)	5601	536 (9.6)	1.1 (1.0–1.3)		1.1 (1.0–1.3)	
**Religion**				<0.001		<0.001
Hindu ®	25144	2112 (8.4)	1		1	
Muslim	2876	327 (11.4)	1.4 (1.2–1.6)		1.4 (1.2–1.6)	
Other	1683	148 (8.8)	1.1 (0.9–1.3)		1.1 (0.9–1.3)	
**Home smoking allowed**				<0.001		<0.001
No ®	27303	2311 (8.5)	1		1	
Yes	2400	276 (11.5)	1.4 (1.2–1.6)		1.3 (1.1–1.5)	
**Family smoking**						
**Father**						
No ®	26881	2286 (8.5)	1	<0.001	1	0.009
Yes	2822	301 (10.7)	1.3 (1.1–1.5)		1.2 (1.1–1.4)	
**Mother**						
No ®	29543	2567 (8.7)	1	0.090		
Yes	160	20 (12.5)	1.5 (0.9–2.4)			
**Siblings**						
No ®	29350	2538 (8.6)	1	0.001	1	0.105
Yes	353	49 (13.9)	1.7 (1.3–2.3)		1.3 (1.0–1.8)	
**Others**						
No ®	25100	2183 (8.7)	1	0.860		
Yes	4603	404 (8.8)	1.0 (0.9–1.1)			
**Friends smoking**				<0.001		<0.001
None ®	26894	2224 (8.3)	1		1	
Anyone	783	133 (17.0)	2.3 (1.9–2.8)		1.8 (1.5–2.2)	
Not sure	2026	230 (11.4)	1.4 (1.2–1.6)		1.3 (1.1–1.5)	
**Smoking is seen in school**				0.007		0.613
No ®	21644	1827 (8.4)	1		1	
Yes	8059	760 (9.4)	1.1 (1.0– 1.2)		1.0 (0.9–1.1)	
**Mother’s education level**				0.150		
No response	415	40 (9.6)	1.2 (0.8–1.8)			
Illiterate	1688	170 (10.1)	1.3 (1.0–1.7)			
Primary	12198	1090 (8.9)	1.1 (0.9–1.4)			
High school	11064	936 (8.5)	1.1 (0.9–1.3)			
Graduate	3115	252 (8.1)	1.0 (0.8–1.3)			
Postgraduate ®	1223	99 (8.1)	1			
**Father’s education level**				0.048		0.239
No response	542	40 (7.4)	0.8 (0.5–1.1)		0.8 (0.5–1.1)	
Illiterate	1232	117 (9.5)	1.0 (0.8–1.3)		0.9 (0.7–1.2)	
Primary	11944	1075 (9.0)	1.0 (0.8–1.1)		0.9 (0.7–1.1)	
High school	11151	962 (8.6)	0.9 (0.8–1.1)		0.9 (0.7–1.1)	
Graduate	3163	235 (7.4)	0.8 (0.6–1.0)		0.8 (0.6–1.0)	
Postgraduate ®	1671	158 (9.5)	1		1	
**Wealth quintile**				0.177		
Lower	5687	508 (8.9)	1.0 (0.8–1.1)			
Lower Middle	6120	519 (8.5)	0.9 (0.8–1.0)			
Middle	5887	504 (8.6)	0.9 (0.8–1.0)			
Middle Upper	6158	506 (8.2)	0.9 (0.8–1.0)			
Upper ®	5851	550 (9.4)	1			
**Rebelliousness**				<0.001		<0.001
No ®	19031	1613 (8.5)	1		1	
Mild	8232	678 (8.2)	1.0 (0.9–1.1)		1.0 (0.9–1.1)	
Moderate	2203	265 (12.0)	1.5 (1.3–1.7)		1.4 (1.2–1.6)	
Severe	237	31 (13.1)	1.6 (1.1–2.4)		1.5 (1.0–2.2)	
**High self-esteem**				0.184		
Strongly agree ®	13816	1186 (8.6)	1			
Agree	6699	577 (8.6)	1.0 (0.9–1.1)			
Neither agree nor disagree	5136	458 (8.9)	1.0 (0.9–1.2)			
Disagree	2258	224 (9.9)	1.2 (1.0–1.4)			
Strongly disagree	1794	142 (7.9)	0.9 (0.8–1.1)			
**School performance**				<0.001		<0.001
Excellent ®	11630	927 (8.0)	1		1	
Good	13868	1201 (8.7)	1.1 (1.0–1.2)		1.1 (1.0–1.2)	
Average	3661	385 (10.5)	1.4 (1.2–1.5)		1.3 (1.1–1.4)	
Below average	544	74 (13.6)	1.8 (1.4–2.3)		1.5 (1.2–2.0)	
**Exposure to tobacco in films**				0.220		0.422^†^
No ®	464	33 (7.1)	1		1	
Yes	29239	2554 (8.7)	1.3 (0.9–1.8)		1.2 (0.8–1.7)	
**Exposure to tobacco intervals in films** (tertiles)				<0.001		0.022^†^
0 ®	464	33 (7.1)	1		1	
1–49	9393	763 (8.1)	1.2 (0.8–1.7)		1.1 (0.8–1.6)	
50–84	10269	838 (8.2)	1.2 (0.8–1.7)		1.1 (0.8–1.6)	
>84	9577	953 (10.0)	1.4 (1.0–2.1)		1.3 (0.9–1.8)	
**Exposure to tobacco in films by COTPA compliance**				0.183		0.132^†^
Watched non-compliant movies	134	7 (5.2)	0.6 (0.3–1.3)		0.6 (0.3–1.2)	
Watched partially compliant movies ®	13294	1126 (8.5)	1		1	

* AOR: adjusted odds ratio; mutually adjusted including age and gender before the addition of film tobacco exposure.

† Smoking in films exposure measures included separately in the adjusted model; mutually adjusted with age and gender, which are common confounding factors along with factors (that are significant in the univariable model with a p<0.05): religion, family smoking - father smoking, friends smoking, rebelliousness, and school performance. ® Reference categories.

Further, in the multinomial logistic regression carried out to study the change in smoking susceptibility as a dependent variable and those who continued to remain non-susceptible as a reference category, it was observed that there was no significant association between watching non-compliant movies and being susceptible (OR=0.6; 95% CI: 0.3–1.4, p=0.223) or changing to being non-susceptible (OR=0.8; 95% CI: 0.4–1.5, p=0.404) compared to children who have watched partially complaint movies.

## DISCUSSION

This is the first individually linked cohort study, to the best of our knowledge, to evaluate the associations between exposure to smoking imagery in films and the onset of susceptibility in Indian adolescents. Our findings suggest tobacco exposure in films increases the risk of incident susceptibility among school children. Aligning with our study, similar results have been reported from longitudinal studies conducted in Argentina^25^ and California^26^. Further, it is also reported that watching movies that were partially compliant with tobacco-free film and TV rules did not have any impact on change in susceptibility to smoking.

The prevalence of susceptibility to smoking at baseline in our study, at 11.8%, was similar to the estimate provided by the Global Youth Tobacco Survey (GYTS) from 168 countries in which 12.5% of adolescents aged 13–15 years were susceptible to smoking^27^. Our finding that susceptibility to smoking was associated with male gender, exposure to parent or peer smoking, and exposure to tobacco advertisements, is consistent with findings from the GYTS and other smaller studies^28,29^ elsewhere in the world. Our study is also similar to another study in which a strong positive association between quartiles of film smoking exposure and susceptibility to smoking is reported^30^. It is also well documented in multiple studies that susceptibility is strongly associated with future smoking^23,31^, indicating that susceptibility in our study is highly likely to identify children at high risk of smoking uptake.

Tobacco-free film and TV rules^12^ were introduced by the Indian government in an attempt to reduce the effect of smoking imagery in films on smoking uptake among young people, although compliance with these rules by filmmakers is partial in most films^24^. Another trend analysis from India has revealed that tobacco depictions in Bollywood films have reduced substantially between 2012 and 2017 from (76% to 35%)^32^. However, changes in the media landscape during the baseline and follow-up of this study would have exposed adolescents to smoking through other media like streaming platforms, as seen in another Indian study that analyzed the content of streaming platforms popular among Indian adolescents and youth^33^. The increasing popularity of online videos and TV series is reflected by 160 million Indian digital video viewers in 2016^34^. Evidence from one study showed that 70% of series portray tobacco use while none was compliant with tobacco-free film and TV rules^33^. This highlights the extensive exposure to tobacco imagery by adolescents through new media.

### Limitations

With the participation of over 80%, our findings are likely to be representative of the population involved. However, this study has some limitations. First, the follow-up period of the study was relatively short but sufficient to analyze the change in susceptibility to tobacco use. Secondly, we have included only film smoking exposure in top-grossing films, while exposure from other films and sources like television programs and online media (which are non-compliant with tobacco-free film and TV rules) may have contributed towards increasing smoking susceptibility as they might tend to influence smoking behavior^35^. Although we had a large sample, the study being conducted in one district might lead to homogeneity of the data, may not reflect the entire population of the country, and may limit the generalizability of study results.

## CONCLUSIONS

Our findings indicate that exposure to smoking imagery in films increases susceptibility to smoking among young people and that the presence or absence of anti-smoking messages or the extent to which films comply with Indian tobacco-free film rules, has little influence on this effect that could be due to high degree of non-compliance to tobacco-free film rules. This suggests that the only way to neutralize the effect of smoking in films is to ensure full implementation of existing rules and minimize exposure. There is also a need to study exposure to smoking imagery in other new media used by adolescents and for more comprehensive approaches to prevent the inclusion of smoking imagery in films.

## Data Availability

The data supporting this research are available from the Principal Investigator on reasonable request.
